# International Conference on Emerging Infectious Diseases 2022 Poster and Oral Presentation Abstracts

**DOI:** 10.3201/eid2809.NN2809

**Published:** 2022-09

**Keywords:** iceid, conference, emerging infections, abstracts

Emerging Infectious Diseases is providing access to these abstracts on behalf of the ICEID 2022 program committee (http://www.iceid.org), which performed peer review. ICEID is organized by the Centers for Disease Control and Prevention and Task Force for Global Health, Inc.

Emerging Infectious Diseases has not edited or proofread these materials and is not responsible for inaccuracies or omissions. All information is subject to change. Comments and corrections should be brought to the attention of the authors.

